# Mean platelet volume is independently associated with systolic and diastolic blood pressure: a retrospective cross-sectional study

**DOI:** 10.1186/s12872-026-06381-9

**Published:** 2026-07-31

**Authors:** Kemal Ozan Lule, Omer Yildirim, Ozge Ozsoy, Hamit Yildiz

**Affiliations:** Department of Internal Medicine, Faculty of Medicine, Gaziantep University, Gaziantep, Türkiye

**Keywords:** Office blood pressure, Elevated blood pressure, Mean platelet volume (MPV), Inflammatory markers, Neutrophil-to-lymphocyte ratio (NLR), ESC 2024, Hypertension

## Abstract

**Background:**

Blood pressure-related cardiovascular risk follows a continuous pattern rather than a dichotomous threshold-based model. In this context, the 2024 European Society of Cardiology (ESC 2024) guidelines emphasize elevated blood pressure as an intermediate-risk category. Low-grade systemic inflammation has been implicated in the pathogenesis of hypertension; however, data regarding inflammatory alterations in individuals with elevated blood pressure remain limited.

**Methods:**

This retrospective cross-sectional study included adults aged 18–65 years attending a general internal medicine outpatient clinic. Participants were classified into three groups based on office blood pressure measurements according to the 2024 ESC guidelines: non-elevated blood pressure, elevated blood pressure, and newly diagnosed hypertension (NDH). Inflammatory and hematological markers, including neutrophil-to-lymphocyte ratio (NLR), platelet-to-lymphocyte ratio (PLR), monocyte-to-lymphocyte ratio (MLR), mean platelet volume (MPV), and C-reactive protein (CRP), were compared across groups. Multivariable linear regression analyses were performed to identify independent associations with systolic and diastolic blood pressure.

**Results:**

A total of 284 individuals were included (mean age 46.7 ± 12.0 years; 65.5% male). Nearly half of the cohort was classified as having elevated blood pressure, while 27.5% had NDH. Age, MPV, and CRP differed significantly across blood pressure categories. MPV was significantly higher in the NDH group and remained independently associated with both systolic and diastolic blood pressure after adjustment for age and NLR. NLR showed weak correlations with systolic blood pressure but was not independently associated in multivariable analyses, while PLR and MLR did not differ significantly across blood pressure categories.

**Conclusion:**

Inflammatory and hematological differences were most evident in newly diagnosed hypertension. Mean platelet volume was independently associated with both systolic and diastolic blood pressure. The temporal and mechanistic significance of this association cannot be determined from this cross-sectional study and requires confirmation in longitudinal research. The 2024 ESC blood pressure classification highlights elevated blood pressure as a clinically relevant intermediate-risk state and underscores the potential value of readily available hematological markers in early cardiovascular risk stratification.

## Introduction

Arterial hypertension is recognized as one of the most important and preventable risk factors for cardiovascular morbidity and mortality [1]. Traditionally, the diagnosis of hypertension has been based on specific thresholds of office blood pressure measurements; however, it has become increasingly evident in recent years that the relationship between blood pressure and cardiovascular risk is linear and continuous. In line with this approach, the 2024 European Society of Cardiology (ESC 2024) guidelines for the management of arterial hypertension have redefined and placed greater clinical emphasis on the ‘elevated blood pressure’ category, which represents the intermediate risk group between normotension and hypertension [2].

According to the ESC 2024 recommendations, individuals with office systolic blood pressure of 120–139 mmHg and/or diastolic blood pressure of 70–89 mmHg are classified as having elevated blood pressure. This category largely corresponds to what earlier guidelines termed prehypertension or high-normal blood pressure. We refer to this earlier terminology because it provides the evidence base demonstrating that this intermediate group already carries an increased cardiovascular risk, a higher likelihood of progression to established hypertension, and a greater burden of subclinical target-organ damage compared with individuals with non-elevated blood pressure [3, 4]. This well-documented risk in the pre-hypertensive range provided the rationale for the present study, in which we examined whether inflammatory activation is already detectable within the ESC 2024 elevated blood pressure category.

Low-grade systemic inflammation is widely recognized as a central contributor to the pathogenesis of hypertension and the development of cardiovascular disease [5]. In this context, inflammatory hematological indices readily derived from the complete blood count, including the neutrophil-to-lymphocyte ratio (NLR), platelet-to-lymphocyte ratio (PLR), monocyte-to-lymphocyte ratio (MLR), and mean platelet volume (MPV), have attracted increasing interest as practical and cost-effective biomarkers associated with endothelial dysfunction, atherosclerosis, and cardiovascular event risk. Similarly, C-reactive protein (CRP), one of the most well-established markers of systemic inflammation, has been shown to be independently associated with both the prevalence of hypertension and adverse cardiovascular outcomes [6–8].

Although numerous studies in the existing literature have demonstrated elevated inflammatory parameters in individuals with established hypertension, data systematically evaluating the inflammatory burden in individuals classified as having elevated blood pressure remain limited. However, early identification of inflammatory activation in this intermediate group may be clinically meaningful for predicting the development of hypertension and associated cardiovascular risks.

Therefore, in this retrospective study, adults attending an internal medicine outpatient clinic were classified into three groups—non-elevated blood pressure, elevated blood pressure and newly diagnosed hypertension (NDH)—based on office blood pressure measurements obtained during routine outpatient visits. The primary objective was to examine the association between office blood pressure categories and systemic inflammatory markers, including the NLR, PLR, MLR, MPV, and CRP. By specifically incorporating the ESC 2024 elevated blood pressure category, this study aimed to determine whether individuals without established hypertension already exhibit evidence of increased inflammatory burden, thereby supporting the concept of elevated blood pressure as an early-risk cardiovascular phenotype.

## Methods

### Study design and population

This study was conducted as a retrospective cross-sectional analysis. The study population comprised patients who were followed at the General Internal Medicine Outpatient Clinic of Gaziantep University Faculty of Medicine between January 2025 and October 2025, and who had office blood pressure measurements recorded during the outpatient visit on the same day that laboratory tests were ordered. Study data were obtained from electronic medical records.

### Inclusion and exclusion criteria

Patients aged 18–65 years who were followed at the General Internal Medicine Outpatient Clinic of Gaziantep University Faculty of Medicine between January 2025 and October 2025 were screened for eligibility. Individuals were included if they had no previously documented diagnosis of hypertension, were not receiving any antihypertensive treatment at the time of evaluation, and had office blood pressure measurements recorded during the outpatient visit on the same day that complete blood count tests were obtained. Participants who met the office blood pressure criteria for hypertension (systolic blood pressure ≥ 140 mmHg and/or diastolic blood pressure ≥ 90 mmHg) at the outpatient visit were classified as having NDH.

Patients were excluded if they were younger than 18 years or older than 65 years. Individuals who had undergone elective or emergency surgical procedures were excluded due to the potential impact of acute physiological stress on inflammatory parameters. Patients whose blood pressure was measured during the outpatient visit but did not have blood samples collected on the same day were also excluded. Additional exclusion criteria included a known diagnosis of diabetes mellitus, evidence of acute infection, or CRP levels greater than 10 mg/L. Patients with a history of chronic inflammatory, autoimmune, hematological, or malignant diseases were not included. Other exclusion criteria comprised pregnancy; regular use of corticosteroids, non-steroidal anti-inflammatory drugs, antiplatelet agents (e.g., aspirin or clopidogrel), or immunosuppressive agents; as well as incomplete, missing, or erroneous laboratory records.

**Fig. 1:**
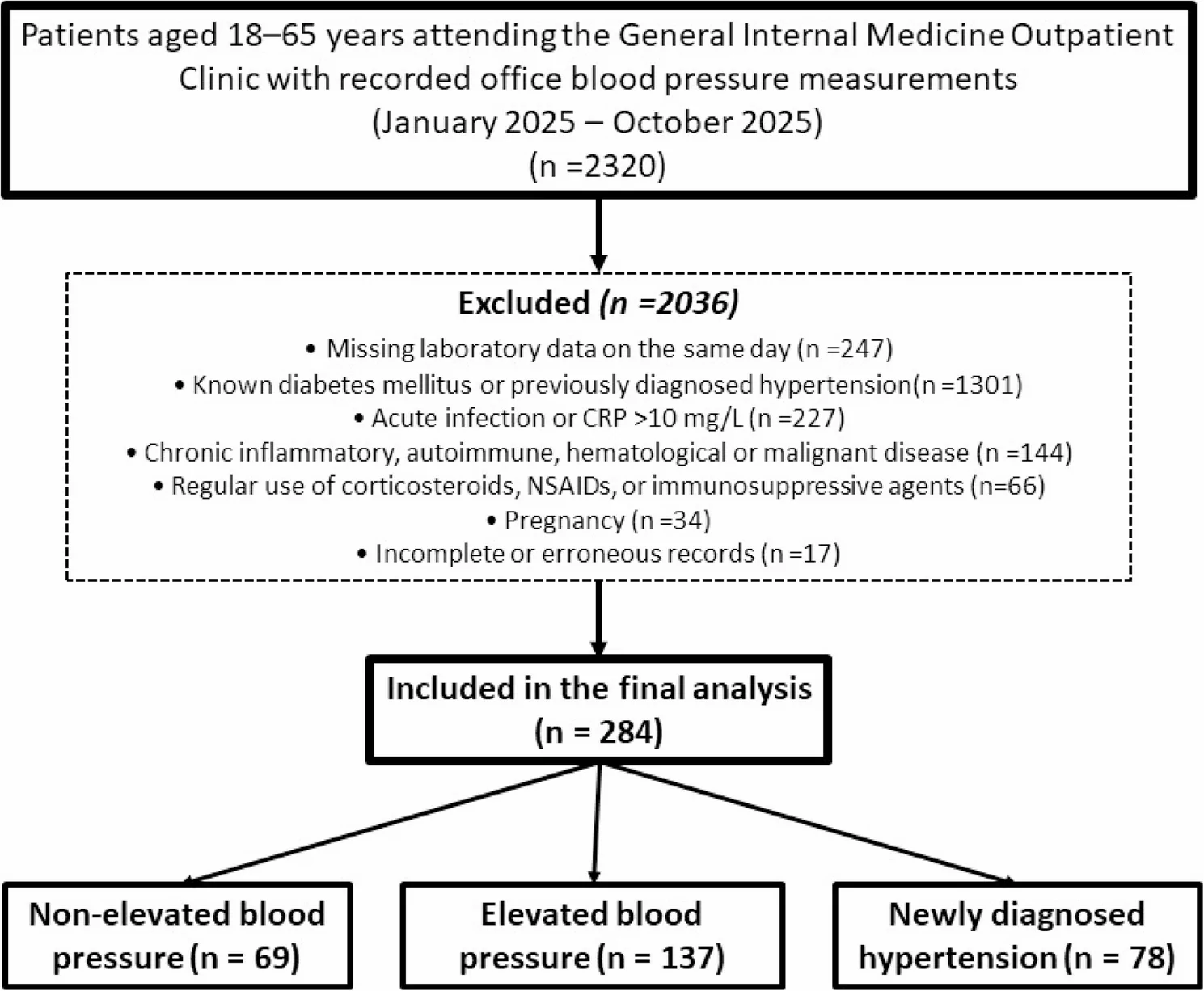
Flowchart of study

A flow diagram illustrating patient selection and exclusion is presented in Fig. 1.

### Blood pressure measurement and group definitions

Office blood pressure measurements were performed in accordance with the ESC 2024 guidelines. At each outpatient visit, blood pressure was measured after a minimum of 5 min of seated rest, using a validated automated oscillometric device and an appropriately sized cuff. Measurements were obtained from the upper arm with the patient in a seated position. Blood pressure was measured three times at 2-minute intervals during the same visit, and the average of the last two measurements was recorded. Based on office blood pressure values, patients were categorized into three groups:

(i) non-elevated blood pressure (systolic blood pressure < 120 mmHg and diastolic blood pressure < 70 mmHg),
(ii) elevated blood pressure (systolic blood pressure 120–139 mmHg and/or diastolic blood pressure 70–89 mmHg), and.
(iii) hypertensive (newly diagnosed; systolic blood pressure ≥ 140 mmHg and/or diastolic blood pressure ≥ 90 mmHg) [2].

### Laboratory measurements

Laboratory data were retrieved retrospectively from the hospital’s central laboratory database. Complete blood count parameters included neutrophil, lymphocyte, monocyte, and platelet counts. The NLR, PLR, and MLR were calculated from these measurements. MPV was recorded as an additional hematological index; the laboratory reference interval for MPV was 9.2–12.2 fL. Biochemical parameters included serum CRP levels. All measurements were originally performed using standardized automated analyzers under internal quality control procedures.

### Sample size and power analysis

Based on the reference study [9], used for sample size estimation, an effect size of ρ = 0.35 was assumed for the association between systolic blood pressure and MPV in patients with hypertension. To detect this correlation as statistically significant, the minimum required sample size was calculated as 59 patients per group (α = 0.05; 1 − β = 0.80). The study was conducted with a total of 284 participants, including 69 individuals with non-elevated blood pressure, 137 patients with elevated blood pressure, and 78 patients with NDH.

### Statistical analysis

Statistical analyses were performed using SPSS version 22.0 (IBM, USA). Categorical variables were expressed as numbers and percentages, whereas continuous variables were presented as mean ± standard deviation along with minimum–maximum values. The distribution of continuous variables was assessed using the Shapiro–Wilk test. Comparisons of normally distributed variables (age, MPV, and CRP) across three groups were performed using one-way analysis of variance (ANOVA), followed by Tukey or Games–Howell post hoc tests, as appropriate. For non-normally distributed variables (NLR, PLR, and MLR), the Kruskal–Wallis test was used, and Bonferroni correction was applied for pairwise comparisons. Associations between continuous variables were evaluated using Pearson or Spearman correlation analysis, depending on data distribution. To evaluate variables independently associated with systolic and diastolic blood pressure, multivariable linear regression analysis was performed using the enter method. Predictors were selected based on clinical relevance and univariable findings: age was included as an established determinant of blood pressure, and MPV and NLR were included as the inflammatory–hematological markers showing significant univariable correlations with blood pressure. Variables without significant univariable associations (PLR and MLR) and CRP were not entered into the models in order to limit the number of predictors relative to the sample size and reduce the risk of overfitting. Results were reported as unstandardized coefficients (B), standardized beta coefficients (β), and 95% confidence intervals. Multicollinearity was assessed using the variance inflation factor (VIF), with a predefined threshold of VIF < 5 adopted to indicate the absence of relevant multicollinearity; all VIF values in the models were < 2, well below this threshold. Model fit was evaluated using the coefficient of determination (R²). The associations of MPV and NLR with systolic and diastolic blood pressure were illustrated using scatter plots with linear regression fit lines. A two-sided p value < 0.05 was considered statistically significant.

In additional binary logistic regression analyses, the adjusted odds of newly diagnosed hypertension versus non-elevated blood pressure were estimated with MPV entered either as tertiles or continuously per 1-fL increase in separate models, with age and NLR included as covariates. Results were expressed as odds ratios (OR) with 95% confidence intervals.

## Results

A total of 284 individuals were included in the study. The mean age of the study population was 46.7 ± 12.0 years, and 65.5% of participants were male. According to the 2024 ESC criteria, nearly half of the cohort was classified as having elevated blood pressure, while 27.5% had NDH (Table 1).

**Table 1:** Baseline demographic, clinical, and laboratory characteristics of the study population

Variable (*n* = 284)	Total population
Age (years)	46.7 ± 12.0
Systolic BP (mmHg)	130.4 ± 15.3
Diastolic BP (mmHg)	76.6 ± 10.2
MPV (fL)	10.31 ± 1.15
CRP (mg/L)	3.88 ± 2.67
NLR	2.02 ± 1.05
PLR	115.7 ± 42.0
MLR	0.25 ± 0.09
Sex, n (%)	
Female	98 (34.5)
Male	186 (65.5)
Office blood pressure categories, n (%)	
Non-elevated blood pressure	69 (24.3)
Elevated blood pressure	137 (48.2)
Newly diagnosed hypertension	78 (27.5)

*Abbreviations*: *BP* blood pressure, *MPV* mean platelet volume, *CRP* C-reactive protein, *NLR* neutrophil-to-lymphocyte ratio, *PLR* platelet-to-lymphocyte ratio, *MLR* monocyte-to-lymphocyte ratio

Data are presented as mean ± standard deviation or number (percentage), as appropriate

Age, MPV, CRP, NLR, PLR, and MLR were compared across office blood pressure categories presented in Table 2 (non-elevated blood pressure, elevated blood pressure according to the 2024 ESC classification, and NDH). Significant differences were observed for age, MPV, and CRP among the three groups. Mean age was significantly higher in the NDH group compared with the non-elevated group (*p* = 0.003), while individuals with elevated blood pressure exhibited intermediate values. Similarly, MPV differed significantly across blood pressure categories (*p* = 0.001), with higher values observed in patients with NDH compared with both non-elevated and elevated blood pressure groups. CRP levels were also significantly elevated in the NDH group relative to the non-elevated group (*p* = 0.029). For non-normally distributed inflammatory indices, NLR demonstrated a significant difference in the overall Kruskal–Wallis analysis (*p* = 0.030); however, pairwise comparisons with Bonferroni correction did not reveal statistically significant differences between individual groups. PLR and MLR did not differ significantly across blood pressure categories (both *p* > 0.05).

**Table 2:** Comparison of demographic and inflammatory parameters across office blood pressure categories

Variable (*n* = 284)	Non-elevated BP (*n* = 69)	Elevated BP (*n* = 137)	NDH (*n* = 78)	Overall *p*-value
Systolic BP (mmHg)	112.9 ± 5.4 ᵃ	127.8 ± 5.2 ᵇ	150.5 ± 9.6 ᶜ	**< 0.001**
Diastolic BP (mmHg)	66.1 ± 3.9 ᵃ	76.3 ± 5.8 ᵇ	86.5 ± 10.5 ᶜ	**< 0.001**
Age (years)	43.1 ± 13.2 ᵃ	46.7 ± 11.6 ᵃᵇ	49.9 ± 10.8 ᵇ	**0.003**
MPV (fL)	10.08 ± 1.38 ᵃ	10.19 ± 1.11ᵃ	10.71 ± 0.88 ᵇ	**0.001**
CRP (mg/L)	3.42 ± 2.62 ᵃ	3.74 ± 2.53 ᵃᵇ	4.53 ± 2.87 ᵇ	**0.029**
NLR	1.75 (1.17–2.17)	1.81 (1.43–2.43)	1.85 (1.45–2.44)	**0.030†**
PLR	106.4 (86.6–133.9)	110.9 (90.8–132.1)	113.9 (90.1–132.1)	0.440
MLR	0.21 (0.19–0.29)	0.23 (0.18–0.30)	0.22 (0.17–0.30)	0.417

Systolic blood pressure, diastolic blood pressure, age, MPV, and CRP were analyzed using one-way ANOVA with Tukey’s HSD or Games–Howell post-hoc tests, as appropriate. Within each row, groups not sharing a common superscript letter (ᵃ, ᵇ, ᶜ) differ significantly from one another (*p* < 0.05) in post-hoc pairwise comparisons, whereas groups sharing the same letter do not differ significantly. Superscript letters are shown only for variables analyzed by one-way ANOVA (systolic blood pressure, diastolic blood pressure, age, MPV, and CRP)

*Abbreviations*: *BP* blood pressure, *NDH* newly diagnosed hypertension, *MPV* mean platelet volume, *CRP* C-reactive protein, *NLR* neutrophil-to-lymphocyte ratio, *PLR* platelet-to-lymphocyte ratio, *MLR* monocyte-to-lymphocyte ratio

Data are presented as mean ± standard deviation for normally distributed variables and median (interquartile range) for non-normally distributed variables

NLR, PLR, and MLR were analyzed using the Kruskal–Wallis test

**†** Overall Kruskal–Wallis test was significant; however, pairwise comparisons with Bonferroni correction did not reveal significant differences between individual groups

Systolic and diastolic blood pressures were strongly correlated with each other (r = 0.783, p < 0.001). Systolic blood pressure showed weak but statistically significant positive correlations with age and MPV, while diastolic blood pressure was also positively correlated with MPV. Among inflammatory markers, only NLR demonstrated a weak but significant correlation with systolic blood pressure, whereas PLR and MLR were not significantly correlated with either systolic or diastolic blood pressure parameters (Table 3).

**Table 3:** Correlations between office blood pressure parameters and clinical and inflammatory variables

Variable (*n* = 284)	Systolic BP	Diastolic BP
Diastolic BP	0.783*	—
Age (years)	0.210*	0.048
MPV (fL)	0.249*	0.202*
CRP (mg/L)	0.104	0.058
NLR	0.120*	0.106
PLR	0.050	0.057
MLR	−0.007	0.048

Pearson correlation coefficients were calculated for normally distributed variables (age, MPV, and CRP). Spearman’s rank correlation coefficients were used for non-normally distributed variables (NLR, PLR, and MLR)

*Abbreviations*: *BP* blood pressure, *MPV* mean platelet volume, *CRP* C-reactive protein, *NLR* neutrophil-to-lymphocyte ratio, *PLR* platelet-to-lymphocyte ratio, *MLR* monocyte-to-lymphocyte ratio

* *p* < 0.05

In multivariable linear regression analyses adjusted for age and NLR, MPV remained independently associated with both systolic and diastolic blood pressure (Table 4). Age was independently associated only with systolic blood pressure, whereas NLR was not independently associated with either systolic or diastolic blood pressure in the adjusted models.

**Table 4:** Multivariable linear regression analyses of systolic and diastolic blood pressure

Variable	Systolic BP B (SE)	95% CI	β	*p*-value	Diastolic BP B (SE)	95% CI	β	*p*-value
Age (years)	0.27 (0.07)	0.13 to 0.41	0.215	**< 0.001**	0.04 (0.05)	−0.05 to 0.14	0.053	0.370
NLR	0.80 (0.83)	−0.82 to 2.42	0.055	0.333	0.28 (0.57)	−0.84 to 1.40	0.029	0.620
MPV (fL)	3.36 (0.75)	1.87 to 4.84	0.252	**< 0.001**	1.79 (0.52)	0.76 to 2.81	0.202	**0.001**
Model R²	**0.112**				**0.045**			
Model p-value	**< 0.001**				**0.005**			

Multivariable linear regression analyses were performed using the enter method. Values are presented as unstandardized coefficients (B) with standard errors (SE), 95% confidence intervals (CI), and standardized beta coefficients (β). Multicollinearity was assessed using the variance inflation factor (VIF), and no significant multicollinearity was detected (all VIF values < 2). Model fit was evaluated using the coefficient of determination (R²)

*Abbreviations*:* BP *blood pressure,* MPV *mean platelet volume,* NLR *neutrophil-to-lymphocyte ratio

In a binary logistic regression analysis comparing newly diagnosed hypertension with non-elevated blood pressure, higher MPV was associated with greater odds of newly diagnosed hypertension after adjustment for age and NLR. Compared with the lowest MPV tertile, the odds were not significantly increased in the second tertile (OR 1.92, 95% CI 0.80–4.57) but were significantly higher in the highest tertile (OR 2.97, 95% CI 1.21–7.30; p = 0.018). When modeled continuously, each 1 fL increase in MPV was independently associated with newly diagnosed hypertension (OR 1.66, 95% CI 1.19–2.31; p = 0.003) (Table 5).

**Table 5:** Adjusted odds of newly diagnosed hypertension (versus non-elevated blood pressure) according to MPV

Variable	OR (95% CI)	*p*-value
MPV tertile		
T1 (7.0–10.0 fL)	Reference	—
T2 (10.1–10.8 fL)	1.92 (0.80–4.57)	0.143
T3 (10.9–13.4 fL)	2.97 (1.21–7.30)	0.018
Age (years, per 1 year)	1.05 (1.02–1.08)	0.002
NLR (per 1 unit)	1.90 (1.18–3.06)	0.009

Binary logistic regression comparing newly diagnosed hypertension (*n* = 78) with non-elevated blood pressure (*n* = 69). In a separate model with MPV modeled continuously, each 1 fL increase in MPV was independently associated with newly diagnosed hypertension (OR 1.66, 95% CI 1.19–2.31; *p* = 0.003)

*Abbreviations*: *MPV* mean platelet volume, *NLR* neutrophil-to-lymphocyte ratio, *OR* odds ratio, *CI* confidence interval

**Fig. 2:**
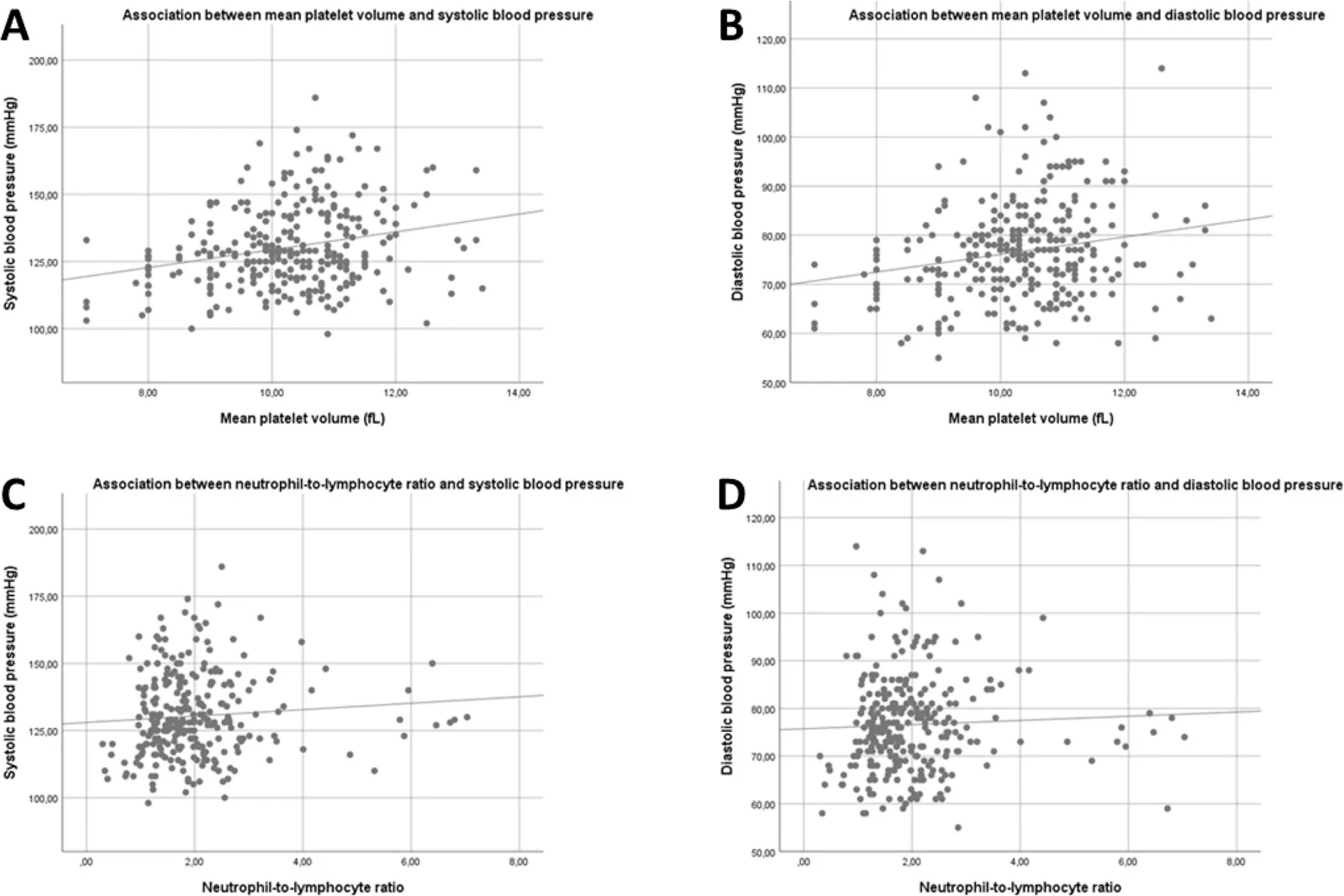
Associations of MPV and NLR with systolic and diastolic blood pressure. Scatter plots illustrating the associations between mean platelet volume (MPV) and neutrophil-to-lymphocyte ratio (NLR) with blood pressure parameters. Panels **A** and **B** show the relationships between MPV and systolic and diastolic blood pressure, respectively. Panels **C** and **D** depict the relationships between NLR and systolic and diastolic blood pressure. Solid lines represent linear regression fits

As shown in Fig. 2, MPV was positively associated with both systolic and diastolic blood pressure, whereas the associations between NLR and blood pressure parameters were weaker and less pronounced.

## Discussion 

In this study, we comprehensively evaluated the associations between office blood pressure categories defined by the 2024 ESC guidelines and a range of demographic and inflammatory parameters in a real-world internal medicine outpatient population. The following discussion contextualizes these findings within the existing literature, with particular emphasis on the clinical relevance of the elevated blood pressure category and the potential role of hematological inflammatory indices in early cardiovascular risk stratification.

The finding that age was higher in the NDH group and remained independently associated with systolic blood pressure is consistent with the well-established concept that advancing age contributes to vascular aging and arterial stiffening, which in turn preferentially elevates systolic blood pressure. Age-associated increases in arterial stiffness have been shown to reduce arterial compliance and augment systolic pressure, reflecting a key pathophysiological link between aging and blood pressure elevation [10, 11].

In the present study, MPV was significantly higher in individuals with NDH and remained independently associated with both systolic and diastolic blood pressure after adjustment for age and NLR. This finding suggests that platelet activation and a prothrombotic–inflammatory milieu are already present at the time of hypertension diagnosis; because disease duration could not be determined, we cannot infer that these changes represent an early stage of hypertension. Our results are consistent with previous prospective evidence demonstrating an independent association between elevated MPV and the development of hypertension, supporting the role of MPV as a marker of cardiovascular risk beyond established blood pressure thresholds [12]. Furthermore, studies conducted in NDH populations have shown that increased MPV is associated with subclinical target organ damage, indicating that MPV may reflect underlying vascular injury rather than merely representing an epiphenomenon [13]. Recent data also suggest that MPV increases in parallel with hypertension severity [14] which aligns with the higher MPV values observed in the NDH group in our cohort. Collectively, these findings support the potential utility of MPV as an accessible hematological marker reflecting platelet-related vascular and inflammatory processes associated with hypertension.

From a pathophysiological perspective, several platelet-related mechanisms may plausibly underlie the association between higher MPV and blood pressure. Larger platelets, reflected by higher MPV, are metabolically more active and exhibit a more pronounced pro-inflammatory and pro-thrombotic phenotype upon activation. Activated platelets release vasoactive mediators such as thromboxane A₂ and serotonin, which promote vasoconstriction and contribute to endothelial dysfunction [15]. In parallel, platelet-derived microparticles and platelet–leukocyte aggregates amplify vascular inflammation and have been implicated in endothelial activation and increased arterial stiffness [16]. Collectively, these pathways provide a biologically plausible basis for the association between MPV and blood pressure observed in our study. Given the cross-sectional design, however, they should be regarded as a mechanistic hypothesis consistent with prior literature rather than a causal or temporal sequence established by our data.

Although NLR initially differed across blood pressure categories, these differences did not remain significant after correction for multiple comparisons. This attenuation of significance indicates that the observed variability in NLR may reflect modest effect sizes, limiting its discriminatory value across blood pressure strata. The weak but significant correlation observed between NLR and systolic blood pressure supports the concept of a continuous relationship between inflammatory activity and blood pressure levels. However, the absence of an independent association in multivariable analyses suggests that this relationship may be largely attributable to shared variability with age or other hematological parameters, rather than reflecting a direct effect of NLR on blood pressure. While large population-based studies have reported an association between NLR and the development of hypertension [17] the magnitude of this relationship appears to vary across study designs and blood pressure phenotypes, particularly in analyses incorporating ambulatory blood pressure measurements [18]. Consistent with this design-dependence, NLR was not independently associated with blood pressure in the continuous linear models but did contribute in the logistic model contrasting newly diagnosed hypertension with non-elevated blood pressure, indicating that the association of NLR with blood pressure is sensitive to the outcome definition and analytic approach.

The higher CRP levels observed in the NDH group compared with the non-elevated blood pressure group suggest that low-grade systemic inflammation may accompany newly diagnosed hypertension. Although CRP is a nonspecific inflammatory marker, its widespread availability supports its potential role as a complementary indicator for early cardiovascular risk stratification [19]. In this regard, the exclusion of patients with CRP > 10 mg/L, together with the exclusion of acute infection and major inflammatory conditions, was intended to minimize the influence of acute inflammation and to better reflect the chronic low-grade inflammatory status relevant to blood pressure elevation.

In our study, PLR and MLR did not differ significantly across office blood pressure categories, suggesting that these indices may be relatively insensitive to blood pressure level alone when assessed using single office measurements. However, evidence from ambulatory blood pressure monitoring studies indicates that PLR may be elevated in specific hypertensive phenotypes, such as non-dipper hypertension [20, 21]. Therefore, the absence of significant differences in PLR and MLR in our cohort may be related to the lack of ambulatory blood pressure phenotyping, which could reveal inflammatory profiles not captured by office blood pressure measurements alone.

The independent association of MPV with both systolic and diastolic blood pressure suggests that platelet activation may parallel overall blood pressure burden, consistent with previous evidence linking increased MPV to hypertension [22]. Consistent with this, participants in the highest MPV tertile had nearly threefold higher adjusted odds of newly diagnosed hypertension than those in the lowest tertile, supporting the potential clinical applicability of MPV as a readily available marker.

Taken together, higher MPV and CRP values were most evident in newly diagnosed hypertension, while the elevated blood pressure group generally showed intermediate values. In particular, MPV was independently associated with both systolic and diastolic blood pressure. Owing to the cross-sectional design, we cannot establish whether this association reflects an early stage of vascular stress; this possibility should be regarded as a hypothesis to be tested in prospective studies. Within this context, the 2024 ESC blood pressure classification offers a clinically meaningful framework by recognizing elevated blood pressure as a distinct intermediate category rather than a benign finding [23]. The substantial proportion of participants classified as having elevated blood pressure in our cohort suggests that readily available hematological markers, such as MPV, may provide complementary information alongside conventional clinical parameters across blood pressure categories.

It should be noted that the multivariable models explained only a modest proportion of blood pressure variability (R² = 0.112 for systolic and 0.045 for diastolic blood pressure). This is expected in cross-sectional observational data, as blood pressure is governed by numerous unmeasured clinical, genetic, and environmental factors. Accordingly, MPV should be interpreted as a complementary marker that may add incremental value within a broader cardiovascular risk-assessment framework, rather than as a standalone predictor of blood pressure or cardiovascular risk.

### Limitations 

Several limitations of this study should be acknowledged. First, the retrospective and cross-sectional design precludes causal inferences between blood pressure categories and inflammatory markers. Second, blood pressure classification was based on office measurements, which may be subject to white-coat or masked hypertension and does not capture ambulatory blood pressure phenotypes such as dipping status. In addition, the newly diagnosed hypertension group was defined by the timing of diagnosis at the index visit rather than by disease onset; because hypertension duration could not be ascertained from the retrospective records, our findings cannot establish whether the observed inflammatory alterations precede or follow the development of hypertension. Third, although major conditions associated with systemic inflammation were excluded, residual confounding by unmeasured factors cannot be entirely ruled out. Finally, this was a single-center study, which may limit the generalizability of the findings to other populations.

## Conclusion

In conclusion, higher MPV and CRP values were most evident in participants with newly diagnosed hypertension, while the elevated blood pressure group generally showed intermediate values. MPV was independently associated with both systolic and diastolic blood pressure. Because of the cross-sectional design, this association should not be interpreted as evidence of early vascular stress, and its temporal and mechanistic significance requires confirmation in longitudinal studies. Consistent with the 2024 ESC blood pressure classification, our findings support the recognition of the elevated blood pressure category as a distinct intermediate-risk state; however, significant pairwise differences in inflammatory and hematological markers between the elevated and non-elevated blood pressure groups were not demonstrated.

## Data Availability

The datasets generated and/or analyzed during the current study are not publicly available due to institutional data protection policies but are available from the corresponding author on reasonable request.

## Abbreviations

ESC 2024: 2024 European Society of Cardiology
BP: Blood Pressure
NLR: Neutrophil-to-Lymphocyte Ratio
PLR: Platelet-to-Lymphocyte Ratio
MLR: Monocyte-to-Lymphocyte Ratio
MPV: Mean Platelet Volume
CRP: C-Reactive Protein
NDH: Newly Diagnosed Hypertension
